# Assessing PET-CT versus conventional CT: a meta-analysis on diagnostic efficacy, prognosis, and post-surgical wound healing in lung mucosal marginal zone lymphoma

**DOI:** 10.3389/fonc.2026.1671661

**Published:** 2026-02-05

**Authors:** Yang Lin, Licai An

**Affiliations:** Department of Hematology, Yantai Yuhuangding Hospital, Yantai, Shandong, China

**Keywords:** computed tomography, diagnosis, lung mucosal marginal zone lymphoma, meta-analysis, positron emission tomography-computed tomography

## Abstract

**Objective:**

This meta-analysis aims to compare the efficacy of positron emission tomography-computed tomography (PET-CT) and conventional CT in assessing the clinical pathological features, prognosis, and post-surgical wound healing in Lung Mucosal Marginal Zone Lymphoma (LMMZL).

**Methods:**

Following PRISMA guidelines, a comprehensive search was conducted across databases including PubMed, Scopus, Web of Science, and the Cochrane Library, culminating in the inclusion of 7 studies representing 353 LMMZL patients. Mean difference (MD) or standardized mean difference (SMD) for continuous data, and risk ratio (RR) or odds ratio (OR) for dichotomous data, were used for the meta-analysis, all with 95% confidence intervals (CIs).

**Results:**

PET-CT demonstrated a superior lesion detection rate (LDR) with an SMD of 7.10 (95% CI: 3.17 to 11.02, *P* < 0.01) and cancer staging detection rate (CSDR) with an SMD of 12.35 (95% CI: 8.99 to 15.7, *P* < 0.01), compared to conventional CT. A significant reduction in false positive rate (FPR) was noted for PET-CT, indicated by an SMD of -44.74 (95% CI: -66.36 to -23.11, *P* < 0.01). Additionally, PET-CT was associated with a notable improvement in wound healing outcomes post-surgery, as evidenced by an SMD of -5.93 (95% CI: -8.69 to -3.17, *P* < 0.01).

**Conclusion:**

PET-CT emerges as a potentially superior diagnostic tool over conventional CT in evaluating the clinical features, prognosis, and post-surgical wound healing in LMMZL. These findings indicated the advanced imaging techniques on enhancing surgical precision and patient recovery in future diagnostic and therapeutic strategies for LMMZL.

## 1 Introduction

Lung mucosal marginal zone lymphoma (LMMZL), a rare entity within the spectrum of primary pulmonary lymphomas, challenges clinicians with its unique presentation and management (1). As a B-cell malignancy predominantly affecting the bronchus-associated lymphoid tissue, LMMZL’s indolent course often masks its potential impact (1). In the realm of diagnosis and treatment, the evolution of imaging techniques, especially positron emission tomography-computed tomography (PET-CT), has been transformative, offering nuanced insights into this complex condition (2).

The fusion of metabolic and anatomical data in PET-CT has been pivotal in refining our understanding of LMMZL (2). This synergy allows for a detailed characterization of the lymphoma, shedding light on its aggressiveness and spread, which is crucial for accurate staging and restaging (3). However, the role of PET-CT extends beyond mere diagnostic capabilities. In cases where surgical intervention becomes necessary, PET-CT’s precision becomes instrumental in guiding surgical strategies, balancing the need for effective tumor removal against the preservation of healthy tissue (4).

While surgery is not a standard approach in managing LMMZL due to its slow progression and response to other treatments, certain clinical scenarios necessitate surgical consideration (5). These situations include localized tumor resections or lymph node dissections, where minimally invasive techniques guided by PET-CT can play a significant role (4). The precision afforded by PET-CT in delineating tumor margins has the potential to minimize surgical trauma, thereby influencing the subsequent wound healing process (4).

This meta-analysis delves into the multifaceted role of PET-CT in LMMZL, with a particular focus on its implications for surgical interventions and post-operative wound healing (6). The analysis aims to unravel how PET-CT-guided surgical decisions impact the trajectory of wound healing, a critical aspect of patient recovery (4). It examines factors such as the extent of tissue resection, the influence of accurate tumor targeting on reducing post-operative complications, and the role of PET-CT in monitoring and managing the wound healing process (4). Furthermore, the analysis seeks to explore the interplay between surgical intervention, wound healing, and long-term outcomes in LMMZL patients.

Given the scarcity of comprehensive studies focusing on post-operative recovery in LMMZL, particularly in the context of wound healing, this analysis aspires to fill this knowledge gap (6). It intends to provide a deeper understanding of how advanced imaging can enhance surgical precision, subsequently affecting wound repair and recovery. This insight is vital for clinicians in optimizing treatment strategies, ensuring minimal invasiveness, and promoting efficient wound healing, thereby improving the overall quality of life for patients with LMMZL.

## 2 Materials and methods

### 2.1 Search protocol

To delve deeper into the role of PET-CT in Lung Mucosal Marginal Zone Lymphoma (LMMZL), we established a comprehensive search protocol. Adhering to PRISMA standards, databases such as PubMed, Scopus, Web of Science, and the Cochrane Library were systematically navigated. Our Keywords incorporated ‘Lung Mucosal Marginal Zone Lymphoma’, ‘PET-CT’, ‘Clinical Implications’, ‘Prognostic Outcomes’, and ‘Post-Surgical Wound Healing’. The search spanned until July 2025, with a preference for articles in English. Preliminary filters were applied based on abstracts and titles, followed by detailed reviews of shortlisted studies.

### 2.2 Inclusion and exclusion criteria

Studies were selected that highlighted both the diagnostic efficacy of PET-CT in LMMZL and the impact of surgical interventions informed by PET-CT on post-operative wound healing. Emphasis was placed on prospective or retrospective cohort study exploring the relationship between PET-CT outcomes and post-surgical recovery, specifically wound healing using the REEDA scale. Excluded were articles not focusing on these aspects, as well as reviews, case reports, and commentaries. Selections were initially made based on titles and abstracts, with full reviews for qualifying studies. Discrepancies were resolved through team discussion.

### 2.3 Data collection

Data extraction was systematic, capturing details such as lead author, study location, publication year, study design, and specific surgical interventions. Demographic data, with a focus on age categories, were noted. The type of PET-CT techniques used, insights on LMMZL prognosis, and details of surgical interventions, including wound healing assessments using the REEDA scale, were meticulously recorded.

### 2.4 Statistical methods

Analysis was facilitated using the Review Manager (RevMan) software, ideal for meta-analytic studies. For continuous data, we used metrics like the mean difference (MD) or standardized mean difference (SMD), each accompanied by 95% confidence intervals (CIs). Dichotomous data were interpreted using risk ratio (RR) or odds ratio (OR) metrics, also with 95% CIs. The I² index was used to measure variability between studies, with a p-value of less than 0.05 denoting statistical significance.

### 2.5 Bias assessment

Given that all the included studies were of an observational design, we used the Newcastle-Ottawa Scale (NOS) to evaluate the methodological quality of the cohort studies. Each study underwent evaluation by two independent reviewers to identify potential biases. Identified biases were then categorized for clarity. To further evaluate publication bias, we used funnel plots, sensitivity analyses, and the Egger’s regression test, ensuring the reliability of our conclusions.

## 3 Results

**Figure 1:**
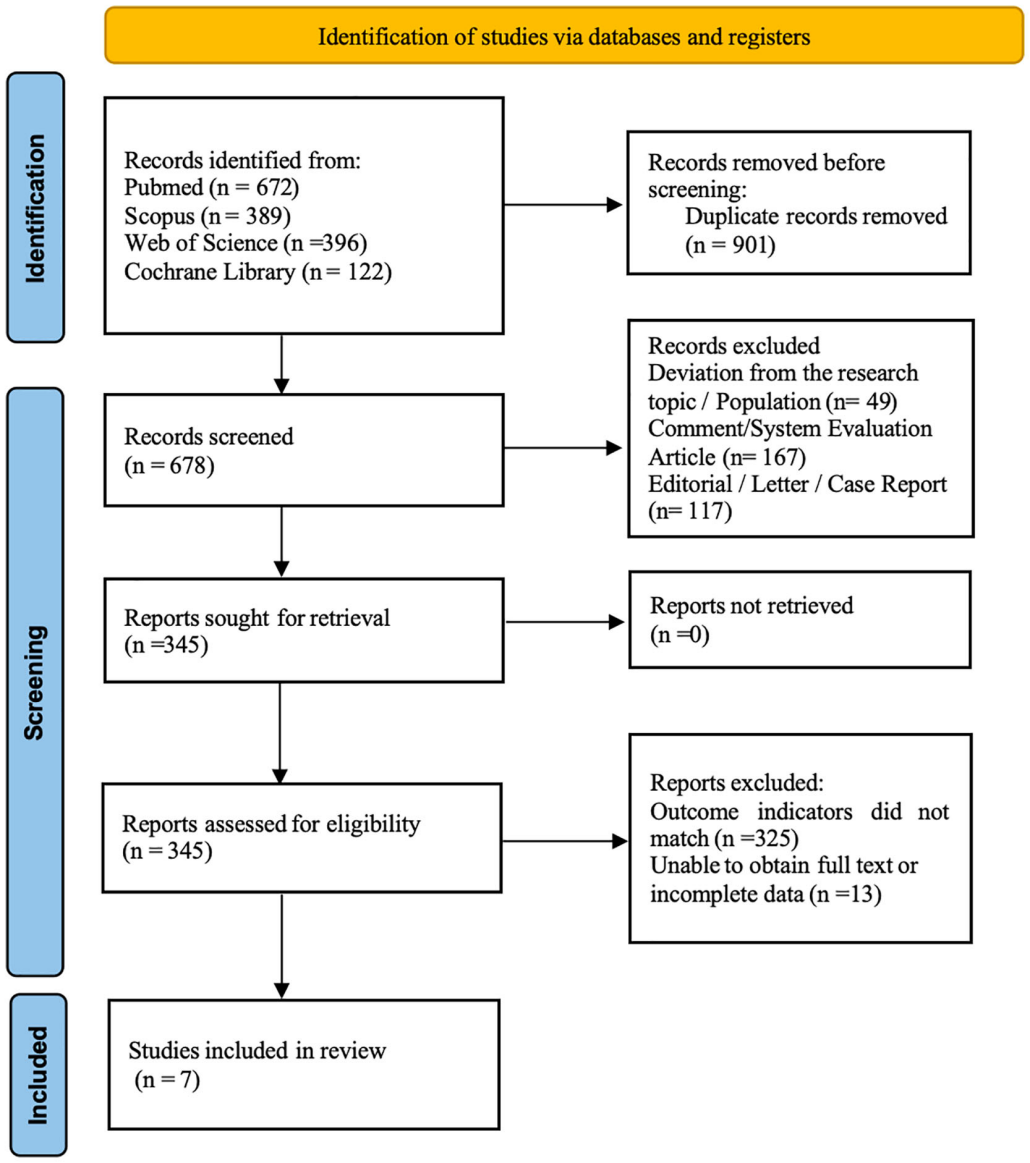
Schematic representation of the literature search strategy and subsequent study inclusion criteria.

Our initial search yielded a total of 1325 articles. After examining duplication, accessibility of full texts, and compliance with the inclusion criteria, 7 studies (2, 7–11) were ultimately selected for inclusion in our meta-analysis, as depicted in **Figure 1**. An updated search conducted up to July 2025 identified 254 additional records. After screening, no new studies meeting the inclusion criteria were found. Among them, 345 studies advanced to the full-text evaluation stage. After a full-text review, we excluded 338 studies. The main reasons for exclusion included: deviation from the research outcome (n=325), and lack of the necessary direct comparison data (n=13). Eventually, 7 studies met all the inclusion criteria and were included in this meta-analysis. The detailed description of the characteristics and interventions of these studies was shown in **Table 1**. These studies encompassed a total of 353 patients diagnosed with Lung Mucosal Marginal Zone Lymphoma, with 182 evaluated using PET-CT and 171 using conventional CT.

**Table 1:** Characteristics of the included observational studies.

First author	Year	Country	Age (years)	Sample size		Measures
			PET/CTRL	PET-CT	CT	
Sheng	2022	China	36.5.2 ± 5.0/42.6 ± 6.3	30	32	Radiological Characteristics (RC), Lymph Node Status (LNS), Extrapulmonary lesions (EL), Overall survival rate (OSR), REEDA, and Disease relapse rate (DRR)
You	2015	Canada	58.14 ± 6.99/50.60 ± 2.07	54	56	RC, LNS, EL, OSR, DRR, Grading of the lymphoma (GL), REEDA, and Progression-free survival rate (PFSR)
Jerusalem	2001	Belgium	47.26 ± 8.10/ 42.15 ± 5.69	20	22	RC, LNS, EL, OSR, GL, REEDA, and PFSR
Zsiray	2009	Hungary	38.5 ± 6.2/30.1 ± 4.2	28	26	RC, LNS, PFSR, GL, REEDA, and OSR
Albano	2017	Italy	45 ± 3/39 ± 2	16	12	RC, EL, OSR, GL, REEDA, and DRR
Zhang	2011	China	*Not Mentioned*	18	12	RC, LNS, GL, REEDA, and OSR
Husnain	2020	USA	51.3 ± 4.7/48.3± 6.3	16	11	RC, LNS, OSR, GL, REEDA, and PFSR

**Table 2** provide an overview of the risk of bias in the selected studies. Overall, in the areas of study subject selection and outcome measurement, the majority of the studies demonstrated low bias risk. However, in terms of group comparability, since all the included studies were of observational design, most of the studies had certain limitations in controlling for known confounding factors, and thus the bias risk in this area was assessed as moderate. Nevertheless, all the studies provided key comparable data.

**Table 2:** Risk of bias assessment of non-randomized comparative studies included in the systematic review.

S. no	Study	Study design	Selection domain	Comparability domain	Outcome domain	Overall score	Risk of bias^#^
1	Sheng 2022	Retrospective cohort study	4	1	3	8	Low
2	You 2015	Retrospective cohort study	4	1	3	8	Low
3	Jerusalem 2001	Retrospective cohort study	3	0	3	6	Low
4	Zsiray 2009	Prospective cohort study	4	1	3	8	Low
5	Albano 2017	Retrospective cohort study	3	0	2	5	Middle
6	Zhang 2011	Retrospective cohort study	3	0	3	6	Low
7	Husnain2020	Retrospective cohort study	3	1	3	7	Low

^#^Risk of bias and quality assessment using New Castle-Ottawa scale.

**Figure 2:**
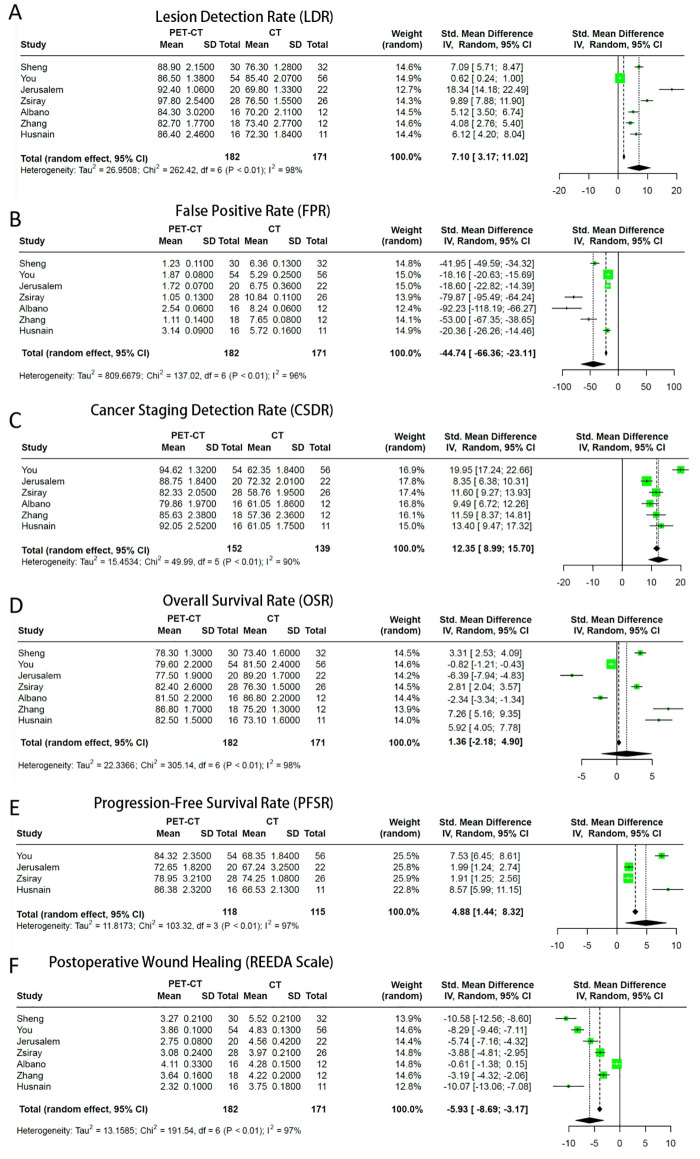
Forest plots comparing outcomes between PET-CT and conventional CT in patients with LMMZL. **(A)** Lesion detection rate. **(B)** False positive rate. **(C)** Cancer staging detection rate. **(D)** Overall survival rate. **(E)** Progression-free survival rate. **(F)** Postoperative wound healing assessed by the REEDA scale.

The forest plot in **Figure 2A**, which compared the lesion detection rate (LDR) between LMMZL patients undergoing imaging with PET-CT *vs*. CT, indicated the detection rate of lesions in the PET-CT group was significantly higher than that in the conventional CT group. (I^2^ = 98%; Random: SMD: 7.10, 95% CIs: 3.17 to 11.02, *P* < 0.01).

Similarly, the false positive rate (FPR) of the PET-CT group was significantly lower than that of the conventional CT group, as illustrated in **Figure 2B** (I^2^ = 96%; Random: SMD: -44.74, 95% CIs: -66.36 to -23.11, *P* < 0.01).

For cancer staging detection rate (CSDR), the results in **Figure 2C** demonstrated PET-CT significantly outperforms conventional CT in the detection rate of cancer staging. (I^2^ = 90%; Random: SMD: 12.35, 95% CIs: 8.99 to 15.7, *P* < 0.01).

The overall survival rate (OSR) comparison in **Figure 2D** indicated no statistically significant difference was observed in the overall survival rates between the two groups. (I^2^ = 98%; Random: SMD: 1.36, 95% CIs: -2.18 to 4.90). Due to the wide confidence interval and the inclusion of the zero value (the null line), it indicates that no statistically significant difference was observed in the overall survival rate between the PET-CT group and the conventional CT group (*P* > 0.05).

Lastly, the progression-free survival rate (PFSR) comparison in **Figure 2E** showed the PET-CT group was significantly higher than that of the conventional CT group (I^2^ = 97%; Random: SMD: 4.88, 95% CIs: 1.44 to 8.32, *P* < 0.01).

All the studies used the REEDA scale to evaluate postoperative surgical site wound healing in patients who underwent surgery for LMMZL. The postoperative REEDA score of patients in the PET-CT group was significantly lower than that of the conventional CT group (I^2^ = 97%; Random: SMD: -5.93, 95% CIs: -8.69 to -3.17, *P* < 0.01), indicating better wound healing in this group. as shown in **Figure 2F**.

**Figure 3:**
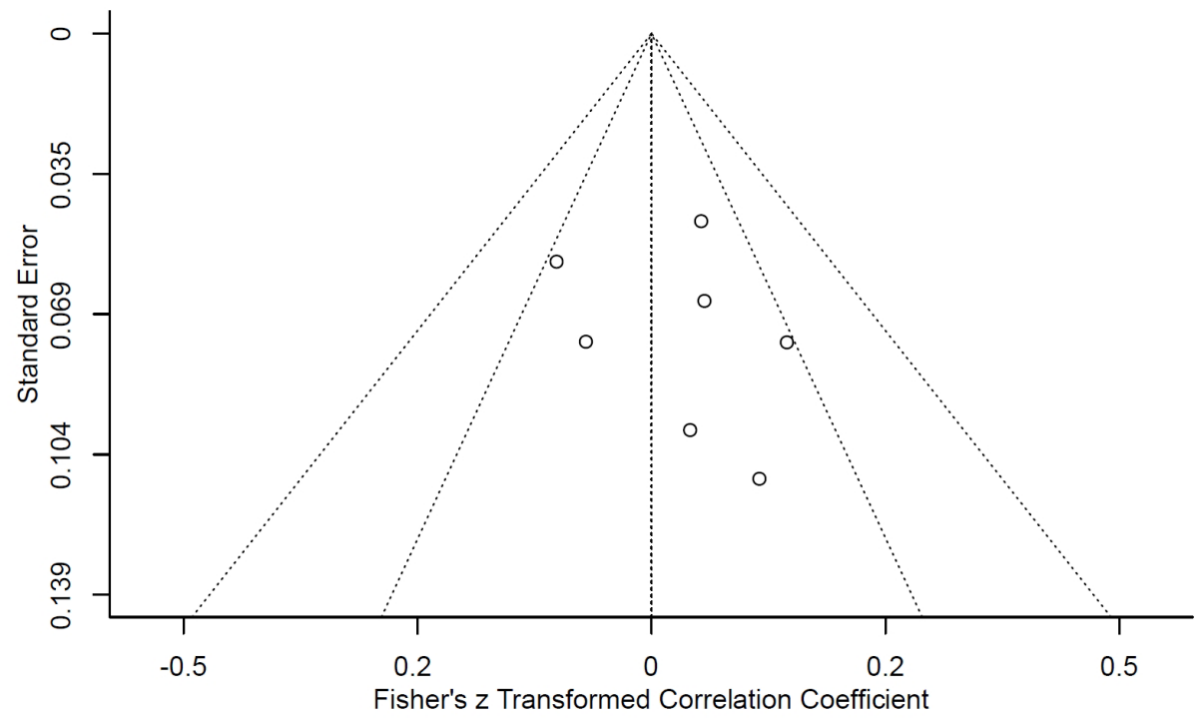
Funnel plot assessing potential publication bias concerning the REEDA between LMMZL patients undergoing imaging pathological examination with PET-CT *vs*. CT.

Regarding publication bias, we specifically assessed the studies involving the REEDA scale for wound healing evaluation using Egger’s quantitative regression analysis and funnel plots. No significant bias was detected in these studies (*P* > 0.05), as shown in **Figure 3**. However, it should be noted that this analysis was limited to the subset of studies that provided data on post-surgical wound healing, and the overall quality of these assessments varied. In addition to the REEDA scale, we also conducted a publication bias assessment for all the primary outcome indicators. **Supplementary Figure S1** shows the funnel plot of all the primary outcome indicators. The funnel plot (**Supplementary Figure S1**) does not show significant asymmetry, but it should be interpreted with caution.

## 4 Discussion

The findings of this meta-analysis illuminate the promising role of PET-CT in the realm of oncological diagnostics, particularly in the context of LMMZL. Beyond its established diagnostic superiority in lesion detection and cancer staging, our analysis reveals an intriguing dimension: the potential influence of PET-CT-guided surgery on post-operative wound healing. The results of this study are mainly based on observational data. Due to the rarity of LMMZL, no randomized controlled trials were included, which may lead to uncontrolled confounding bias. Nevertheless, these preliminary findings provide important references for the potential advantages of PET-CT.

PET-CT’s precision in surgical planning is not only instrumental in enhancing lesion detection but also plays a crucial role in optimizing surgical outcomes. This is particularly evident in our analysis of wound healing, where PET-CT guidance appears to contribute to improved post-operative recovery. The use of PET-CT in surgical planning may facilitate more accurate tumor resection, minimizing unnecessary tissue damage and potentially leading to better wound healing outcomes, as suggested by the trend in our data (12, 13). However, the causal direction of this association must be interpreted with caution. Firstly, all the included studies did not directly compare a randomized controlled trial scheme where PET-CT was applied in surgical planning versus when it was not. Therefore, the observed association is based on the comparison of patient groups with different initial imaging assessments. Secondly, this association may be influenced by multiple important confounding factors, such as: patients who chose to undergo PET-CT may have different clinical characteristics or tumor biological behaviors; the centers that used PET-CT may have overall more advanced surgical techniques and perioperative management; there may be differences in specific surgical methods, resection range, and adjuvant therapy between the PET-CT group and the non-PET-CT group. All these factors may independently affect the wound healing outcome and thereby cause confusion with the use of PET-CT (14, 15).

Despite the less pronounced difference in overall survival rates between PET-CT and conventional CT, our findings regarding the improvement in progression-free survival rates with PET-CT underscore the potential for more effective, targeted therapeutic interventions. This could, in turn, positively influence post-surgical recovery and wound healing processes (16–19).

This study found that PET-CT did not bring about statistically significant overall survival benefits (SMD: 1.36, 95% CI: -2.18 to 4.90). This result is highly consistent with the inherent indolent biological behavior of pulmonary mucosa marginal zone lymphoma (LMMZL). LMMZL is a slow-growing low-grade B-cell lymphoma, and patients usually have a good prognosis, with a median survival period often exceeding 10 years. In this disease with a naturally long course, any improvement in a single diagnostic or staging technique may bring about survival benefits that are difficult to detect within a relatively limited follow-up period (in most studies of this meta-analysis, the follow-up period may not be sufficient to cover the entire survival trajectory of the disease). More accurate staging (such as that shown by PET-CT) mainly affects risk stratification and initial treatment decisions (for example, differentiating between the localized and disseminated stages), but for this type of indolent lymphoma, various strategies such as observation, local treatment, or systemic treatment may all yield similar long-term survival outcomes. Therefore, an improvement in diagnostic accuracy may not translate into distinguishable survival curves between different imaging-guided strategies.

Although no direct impact on overall survival rate was found, this does not mean that PET-CT lacks prognostic value in the long-term management of LMMZL. Its value may be manifested in the following aspects: First, precise staging and risk redefinition: PET-CT more accurately identifies hidden extranodal or lymph node involvement, and can “upgrade” some patients originally classified as limited-stage by conventional CT to a more advanced disease stage. This avoids unnecessary and invasive local treatments (such as surgery) for patients who are actually in the disseminated stage, thereby possibly protecting the patient’s quality of life and guiding them to receive more appropriate systemic treatment. Second, treatment response assessment and early recurrence monitoring: PET-CT is more sensitive than CT in the evaluation of treatment efficacy (such as Deauville score) after the end of treatment, and can detect treatment resistance or micro-residual lesions earlier and more reliably. This allows clinicians to promptly adjust treatment strategies, which may help delay disease progression and perhaps explain the significant advantage of the PET-CT group in progression-free survival (PFS) observed in this study. Third, as an alternative biomarker of biological invasiveness: Metabolic parameters such as SUVmax in PET-CT may reflect the proliferative activity of the tumor and can serve as biomarkers for predicting disease transformation or more aggressive clinical processes, assisting in individualized follow-up and intervention. Therefore, the “prognostic value” of PET-CT should be understood as improving the quality of treatment decisions and the patient’s progression-free survival through optimizing risk stratification, guiding precise treatment, and providing more sensitive efficacy monitoring, rather than directly and independently extending the overall survival time of all patients. In tumors like LMMZL that are inert, the latter are influenced by multiple complex factors and require extremely long-term follow-up to reveal differences.

In conclusion, while PET-CT undoubtedly enhances diagnostic accuracy in LMMZL, its impact on post-surgical wound healing is an area ripe for further investigation. Our study highlights the need for more focused research to fully understand the extent to which PET-CT can influence surgical outcomes and patient recovery in LMMZL, particularly in terms of wound healing and long-term quality of life (20, 21).

PET-CT stands at the forefront of a diagnostic revolution in oncological care, particularly in LMMZL assessment. Our findings suggest that its impact extends beyond mere diagnostic accuracy to potentially influence surgical outcomes and post-operative wound healing. As we continue to delve into the capabilities of PET-CT, a comprehensive approach encompassing accurate diagnosis, targeted therapeutic interventions, and optimized post-surgical care becomes essential.

This meta-analysis observed extremely high statistical heterogeneity (I² > 90%), which severely limited the interpretability of the combined effect size. This heterogeneity may stem from multiple aspects (1): Clinical heterogeneity: including differences in patient populations regarding LMMZL stage, physical condition, and comorbidities; differences in surgical techniques (VATS *vs*. open surgery) and PET-CT scanning protocols. (2) Methodological heterogeneity: variations in study design (prospective *vs*. retrospective), the evolution of CT techniques in the control group, and the limitations in the validity of using the REEDA scale to assess chest surgical wounds. These factors collectively led to significant variations in the estimated effect values of each study.

Our meta-analysis, while insightful, has several inherent limitations. The main limitation lies in the fact that all the included studies were of observational design, which inherently carry risks of selection bias and confounding bias. Although we attempted to control for heterogeneity through rigorous bias assessment and random effect models, the interpretation of the results still requires caution. Secondly, the focus of most studies on the diagnostic aspects of PET-CT, rather than directly on surgical outcomes and post-operative healing, limits the depth of our conclusions in these areas. Despite efforts to mitigate publication bias, the relatively small number of studies specific to PET-CT in LMMZL and wound healing could skew our analysis. Thirdly, advancements in PET-CT technology and evolving clinical practices may limit the applicability of our findings over time. Therefore, while our study sheds light on important aspects of PET-CT in LMMZL, it underscores the need for ongoing research and more targeted studies to bolster these preliminary insights. Fourthly, there are significant limitations in the outcome assessment tools. All included studies used the REEDA scale to evaluate postoperative wound healing. This scale was originally designed for perineal wounds, and its effectiveness and reliability for chest surgery incisions (such as VATS or open thoracotomy) have not been fully validated. Although it provides a structured assessment of local inflammatory signs (such as redness and exudation), it may not fully capture the unique dimensions and complications of chest wound healing. Therefore, the conclusion that the PET-CT group had better wound healing based on the REEDA scale should be regarded as a preliminary indication. The clinical significance of this conclusion needs to be confirmed through future studies using dedicated wound assessment tools specifically validated for chest surgery (such as: patient-reported outcomes, imaging assessment, or comprehensive complication index assessment). Fifthly, although we identified the potential clinical and methodological sources, due to the limited number of included studies (n=7) and insufficient reporting details, we were unable to conduct the planned subgroup analysis or meta-regression to explore the impact of specific factors (such as different surgical methods or PET-CT protocols) on the results. Therefore, the combined effect size obtained in this study should be regarded as a rough and exploratory estimate of the true effect, rather than an exact summary. This high heterogeneity situation precisely highlights the urgency of future research on standardized imaging assessment and surgical reporting of LMMZL, in order to conduct more meaningful comprehensive comparisons. Sixthly, there are fundamental limitations in the inference regarding the impact of PET-CT on wound healing. The core issue lies in the lack of prospective evidence for a direct comparison between PET-CT-guided and non-PET-CT-guided surgeries. The observed healing advantage correlation cannot be established as a causal relationship. The most reasonable explanation is that PET-CT, as a more advanced diagnostic tool, indicates the use of a comprehensive treatment path that includes more comprehensive evaluations, potentially better surgical decisions, and superior perioperative care. The improvement in wound healing is likely the result of this overall ‘package effect’ rather than the direct product of PET-CT imaging guidance. Future rigorous studies (such as randomly assigning patients who have undergone PET-CT staging to groups with or without surgical planning based on or without the information from this PET-CT) are needed to test this hypothesis. Seventhly, in this study, some of the SMD values were relatively high, suggesting that PET-CT has significant advantages in several indicators. However, when interpreting these results, the characteristics of the indicators, the internal variation of the study, and the influence of the combination method should all be taken into account. Future research can further verify these findings through meta-analysis of individual patient data or by using ratio-based effect sizes. Finally, the literature search strategy has limitations, which may increase the risk of publication bias. Our search was mainly limited to English-published literature, and did not systematically cover grey literature sources. This means that relevant studies published in other languages or unpublished negative results may have been missed, thereby potentially overestimating the effect size of PET-CT. Although we conducted a publication bias test for the primary outcome and found no significant evidence, in the context of a small number of studies, the power of these tests is limited and cannot completely rule out the possibility of publication bias. Although we extended our systematic literature search to July 2025 to capture the most recent evidence, no additional eligible studies were identified beyond those included in the initial search. This underscores the ongoing scarcity of comparative studies on PET-CT in LMMZL but also confirms that our analysis represents the current body of evidence up to mid-2025.

## Data Availability

The original contributions presented in the study are included in the article/**Supplementary Material**. Further inquiries can be directed to the corresponding author.
