# Supplementary figures and images for: Assessing PET-CT versus conventional CT: a meta-analysis on diagnostic efficacy, prognosis, and post-surgical wound healing in lung mucosal marginal zone lymphoma
Source: Front Oncol. 2026 Feb 5;16:1671661. doi: 10.3389/fonc.2026.1671661 (PMC12916363; doi:10.3389/fonc.2026.1671661)

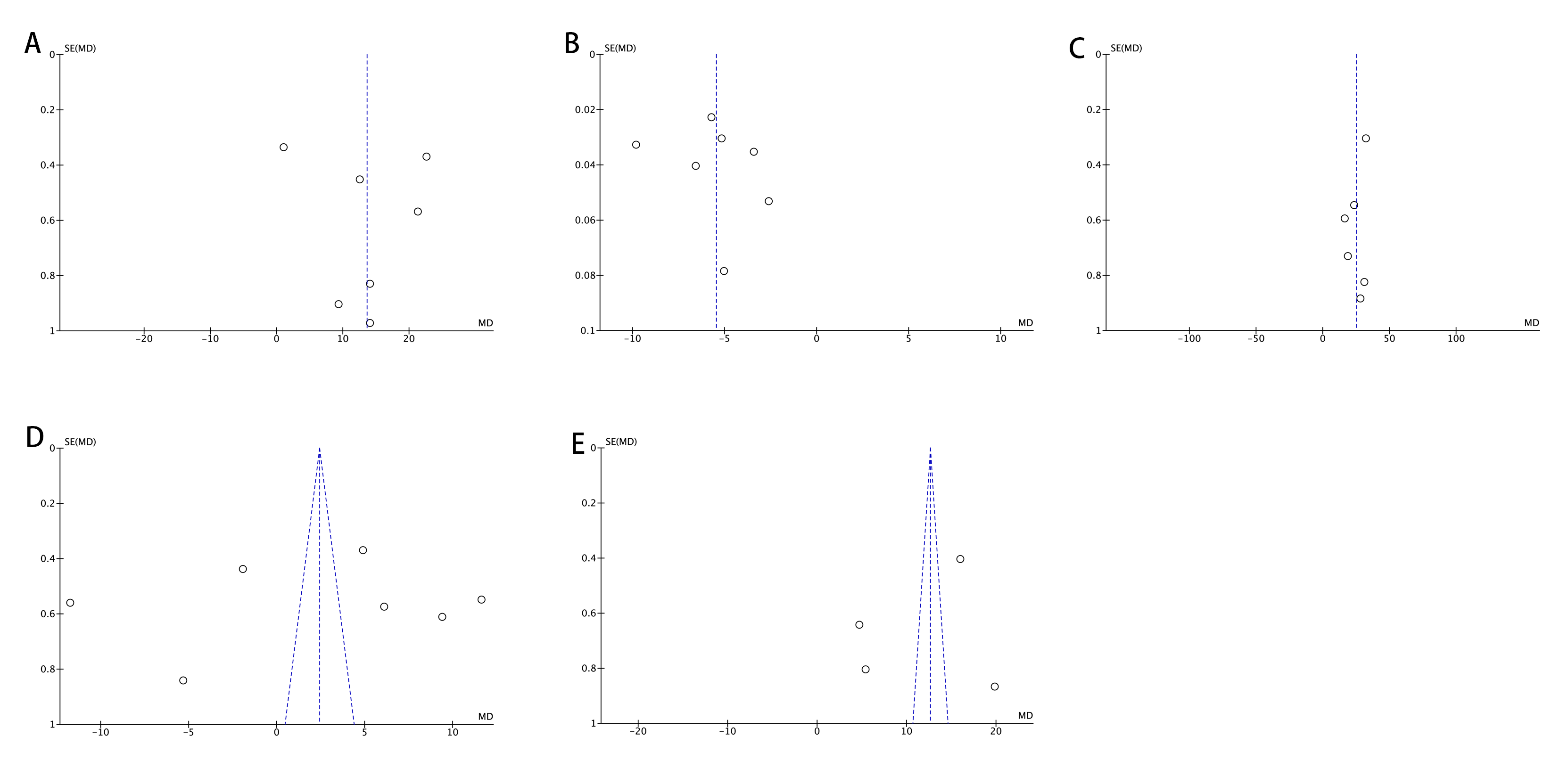

Supplement: Supplementary file 1 [file Image1.tif]
